# Advances in Immune Microenvironment and Immunotherapy of Isocitrate Dehydrogenase Mutated Glioma

**DOI:** 10.3389/fimmu.2022.914618

**Published:** 2022-06-13

**Authors:** Dongming Yan, Weicheng Li, Qibing Liu, Kun Yang

**Affiliations:** ^1^ Department of Neurosurgery, The First Affiliated Hospital of Hainan Medical University, Haikou, China; ^2^ Department of Pharmacology, School of Basic Medicine and Life Sciences, Hainan Medical University, Haikou, China; ^3^ Department of Pharmacy, The First Affiliated Hospital of Hainan Medical University, Haikou, China

**Keywords:** glioma, isocitrate, dehydrogenase, tumor microenvironment, immunotherapy

## Abstract

The tumor immune microenvironment and immunotherapy have become current important tumor research concerns. The unique immune microenvironment plays a crucial role in the malignant progression of isocitrate dehydrogenase (IDH) mutant gliomas. IDH mutations in glioma can inhibit tumor-associated immune system evasion of NK cell immune surveillance. Meanwhile, mutant IDH can inhibit classical and alternative complement pathways and directly inhibit T-cell responses by metabolizing isocitrate to D-2-Hydroxyglutaric acid (2-HG). IDH has shown clinically relevant efficacy as a potential target for immunotherapy. This article intends to summarize the research progress in the immunosuppressive microenvironment and immunotherapy of IDH-mutant glioma in recent years in an attempt to provide new ideas for the study of occurrence, progression, and treatment of IDH-mutant glioma.

## Introduction

According to the 2016 WHO revised categorization of central nervous system malignancies and recognized histological characteristics associated with the natural course of the disease, diffuse oligodendrocytes and astrogliomas in adults are rated from grade II to grade IV (1). The presence or absence of isocitrate dehydrogenase (IDH) mutation and chromosomal co-deletion of 1p/19q are used to further classify these malignancies (1, 2). Adult gliomas are classified into three groups: IDH-mutated tumors, 1p/19q co-deletion tumors, and primary oligodendrogliomas. IDH-mutated tumors are associated with a favorable prognosis; non-1p/19q co-deletion tumors and primarily astrocytic gliomas are associated with a moderate prognosis; and IDH wild-type tumors and primarily WHO grade III or IV glioblastomas are associated with a poor prognosis (3). Although most glioblastomas belong to the IDH wild type, IDH mutant glioblastomas have now been identified as a separate disease entity linked to IDH mutant WHO II and III gliomas.

IDH-mutant astrocytomas and oligodendrogliomas (IDH-As and IDH-Os) share the same developmental hierarchy and lineage of glial differentiation, and the volume differences between them can be largely explained by discrete TME and landmark genetic events, as tumor grade increased, malignant cell proliferation was enhanced, undifferentiated glioma cells increased, and macrophage expression programs overtook microglia in the TME (4). IDH-A is characterized by a loss of function in ATRX that is essentially universal (inactivated in 86 percent of IDH-A). However, in IDH-O, ATRX mutations are uncommon. Comparing IDH-A and IDH-O cells of the same type, there are considerable changes in transcription factor expression and targeting (5). More than 70% of the WHO grade II and III astrocytomas, oligodendrogliomas, and glioblastomas originating from these low-grade lesions have mutations affecting amino acid 132 of IDH1, and alterations affecting IDH2-like amino acids R172 are common in tumors without IDH1 mutations (6). IDH mutations linked to cancer are most commonly found in the arginine residue, which is required for isocitrate recognition (R132 for IDH1, R140, or R172 for IDH2). Isocitrate is effectively converted to D-2-hydroxyglutarate (2-HG) by mutant IDH, resulting in exceptionally high concentrations in TME (7, 8). Tumor-derived 2-HG may operate as an intercellular mediator in TME, affecting non-tumor cells.

Immune surveillance relies heavily on the immune system. The immune system can continuously monitor abnormal cells in the body, identify, and destroy them. Immune cells in brain tumors typically include NK cells, T lymphocytes, dendritic cells (DCs), and microglia (9). Tumor-infiltrating immune cells are part of the complex tumor microenvironment (TME) and may play a significant role in either preventing or promoting tumor growth. Tumorigenesis is characterized by immune evasion, which is a significant obstacle to effective treatment for cancer (10). Although significant progress has been made in tumor immunotherapy approaches and clinical efficacy over the previous years, the association between IDH mutations and a better prognosis in glioma remains largely unknown. IDH may be an ideal target for targeted therapy and is expected to become the preferred target of immunotherapy, thus providing a new perspective for the clinical treatment of glioma. Much of the discussion below is primarily related to the immune microenvironment and immunotherapy in IDH-mutant gliomas.

## The Immune Microenvironment of Isocitrate Dehydrogenase Mutant Gliomas

### IDH Mutations Suppress the Tumor-Associated Immune System

In the process of tumorigenesis and malignant progression, tumor-infiltrating immune inflammatory cells play an important role. Once recruited into the tumor microenvironment, these cells can promote the malignant progression of the cancer cell phenotype. In addition, they also establish a complex network of cell-to-cell interactions, which help to improve and maintain the immunosuppressive microenvironment, promote immune escape, and ultimately promote the development of tumors. Immune infiltration is lower in Mutant IDH1/2 gliomas than in wild-type IDH1/2 gliomas (11–13). IDH-As are more heavily infiltrated by monocytic-lineage cells derived from circulation, they upregulate myeloid-cell chemotaxis genes (CSF1, FLT3LG) and upstream transcription factors (NFKB1), compared to IDH-Os (5). Moreover, immune cell (including M0, M1, and M2 macrophages) infiltration in WHO II IDH-As was generally higher than in WHO II IDH-Os, whereas T cells (CD3+, CD4+, and CD8+), cytotoxic cells, and T helper cells infiltrated in IDH-Os were significantly higher than those in IDH-As (14, 15). Reduced tumor purity (i.e., increased invasion of glioma by non-tumor cells such as immune and stromal cells) was linked to higher malignancy and shorter survival (16). A significant increase in T cells (CD3+), cytotoxic cells, and T helper cells infiltration were observed in recurrent IDH-mutant gliomas compared with primary IDH-mutant tumors, while the opposite was observed for regulatory T cells, this change was associated with prior or not radiotherapy (14). CCL-2 is thought to attract white blood cells (monocytes, memory T cells, and DCs) to inflammatory areas caused by tissue damage or infection, CXCL-2 has chemotactic effects on neutrophils, monocytes, and macrophages (17, 18). C5a is a mediator of chemotaxis and cellular release reactions, playing an essential role in innate immunity and adaptive immunity as well (19, 20). Cytokine arrays revealed that the three genes mentioned above were down-regulated at the mRNA and protein levels in IDH1-mutated tumors, suggesting that immune infiltration and chemotaxis are regulated by IDH1 mutations and that a reduced aggressive part of the tumor-associated immune system could provide IDH-mutant glioma patients with a longer survival duration (12).

### IDH Mutations Evade NK Cell Immune Surveillance

The accumulation of natural killer cells and regulatory T cells leads to leukopenia and immune impairment (21). Natural killer (NK) cells serve as the main effector cells of cancer in innate immunity and are the first line of defense against diseases including malignancies. To evade NK cell-mediated immunity, malignant tumors employ a variety of tissue and mutation-specific strategies. NK cell activity is regulated by the combination of signals that activate and inhibit NK cell surface receptors (22). The activation of NK cells leads to the release of cytotoxic granules containing perforin, various granzymes, and cytokine production, most prominently IFN-γ (23, 24) Killer cell Lectin-like receptor K1 (also known as NKG2D, KLR) is an activated NK cell and CD8+ T cell receptor that mediates cytotoxicity by attaching stress-induced ligands to target cells. MHC class I associated chains A and B (MICA, MICB) and the UL16-binding protein family (ULBP1-6) are among the NKG2D ligands (NKG2DLs) (25). The expression of NKG2DLs in tumor cells is induced by oncogenic stress caused by genetic instability or metabolic derangement, and the expression of these ligands may be essential for antitumor immunity during the elimination phase of innate immune tumor surveillance (26, 27). Following activation of NKG2DLs, NKG2D-activated receptors enhance cytokine production and perforin-mediated cytotoxicity (28), when the expression of NKG2DL in tumors is proportionate to NK-mediated cytotoxicity (29).

**Figure 1:**
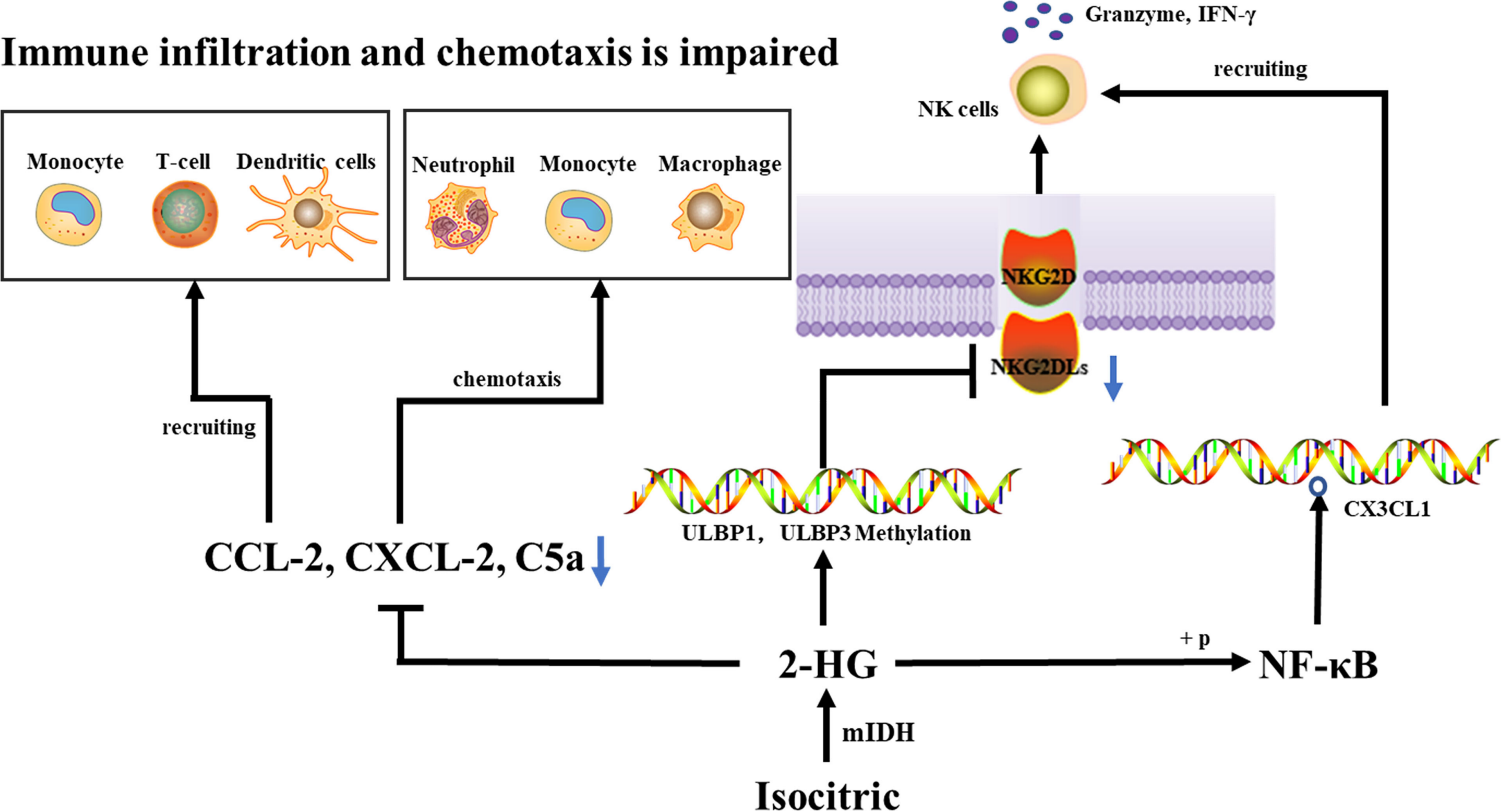
An IDH1 mutation lowers the expression of CCL-2, CXCL-2, and C5a, which reduces immune cell recruitment and chemotaxis, thus inhibiting the tumor-associated immune system. IDH mutations cause hypermethylation of the ULBP1 and ULBP3 promoters, as well as a decrease in NKG2DL, which affects NKG2DL-NKG2D interaction, inhibits NK cell activation, and ultimately escapes immune surveillance by NK cells. 2-HG can activate NF-κB, regulate CX3CL1 expression, and then CX3CL1 recruit NK cells to the tumor location.

ULBP1 and ULBP3 are MHC class I proteins bound through the glycosylphosphatidylinositol anchor membrane (30). Both ligands are members of the UL16 bound NKG2DLs with 6 members (ULBP_1-6_) (31). NKG2D receptors on NK cells identify membrane-bound ULBP1 and ULBP3, and these ligands activate NK cells and cause target cell lysis through NKG2D binding (26). IDH wild-type tumors had significantly high levels of NKG2DLs, ULBP1, and ULBP3, while IDH mutant tumors did not. the promoter methylation levels of ULBP1 and ULBP3 were higher in IDH-mutant tumors than in IDH wild-type tumors, ULBP1 and ULBP3 were transcriptionally silenced when their promoters were hypermethylated, thus decreasing the NKG2DL expression, which in turn affected NK cell activation and enhanced resistance to NK-mediated cytotoxicity (32), thereby evading immune surveillance by NK cells. Interestingly, in IDH1-mutant gliomas, 2-HG can activate NF-κB, regulate CX3CL1 expression, and then CX3CL1 recruits NK cells to the tumor location (5, 33). Moreover, decreased MHC-I expression in IDH-mutant gliomas is associated with higher DNA methylation levels of MHC-I HLA genes (34). Reduced expression of HLA class I molecules leads to upregulation of activated NK receptor recognized ligands, facilitating NK cells mediated lysis (35) (**Figure 1**).

### 2-Hydroxyglutarate Inhibits Classical and Alternative Complement Pathways

Complement is an important aspect of immune surveillance mechanisms in host tumor cells but may play an opposite role in carcinogenesis, as activating complement causes inflammation, which promotes tumor development. Complement on tumor cells could indeed be directly activated by tumor cells themselves (36, 37), or by tumor-reactive antibodies that attach to neoantigens on the surface of tumor cells, allowing membrane attack complex (MAC) mediated lysis and boosting tumor phagocytosis (38).

C3b (iC3b) was deposited on target cells after complement activation, facilitating phagocytosis (39). C3(C3b) was deposited on the surface of IDH wild-type and mutated glioma cells. Compared with IDH wild-type gliomas, IDH mutants tend to have lower complement depositing on the surface of tiny blood vessels and capillaries. IDH mutant glioblastoma had lower levels of C3 (C3b) deposition in the necrotic area as compared with IDH wild-type glioblastoma. 2-HG significantly reduced C3 deposition on cells through a dose-dependent approach, inhibited C3b (iC3b) complement-mediated phagocytosis and opsonization, and inhibited the assembling of C5 but not C3 convertases in the classical complement activation pathway. Complement-mediated cellular damage is inhibited at numerous phases of the complement activation process (40), rather than inhibiting complement activity through simple calcium linkages (41). The alternate complement route is a separate main complement activation mechanism that amplifies complement activation triggered by other pathways. C3b is required with both C3 but also C5 convertases in the alternate pathway. The assembling of C3/C5 conversion enzymes in the complement activation alternative route was inhibited by 2-HG, while the activity of prebuilt C3/C5 conversion enzymes in the complement activation alternative route was not significantly affected. During pre-assembly and assembly, there was a biochemical and functionality transition that occurs. Complement activation was inhibited by 2-HG, which reduces MAC-mediated brain cancer cell damage (40).

### 2-Hydroxyglutaric Acid Directly Inhibits T Cell Responses

Tumor-reactive T cells, which are triggered by tumor antigen-presenting cells like DCs, multiply and release cytotoxin-like granzymes and create inflammatory cytokines like IFN-γ, playing a crucial role in tumor immune surveillance (42, 43). T cells also stimulate humoral responses, inducing the production of tumor-specific antibodies to activate complement on tumor cells, resulting in the assembly of MAC pores, lysis, identification, and phagocytosis by giant cells. T-cell factors are a core component of acquired immunity, and DCs plays a key role in T-cell activation. In primary IDH1-R132H grade II/III gliomas, less infiltration of CD8^+^ T cells in the TME is associated with long-term survival (11, 44). Extensive infiltration of Treg cells into tumor tissue is often associated with poor prognosis in cancer patients, and Treg cell depletion enhances antitumor immune responses, but may also trigger autoimmunity (45). Studies demonstrated that 2-HG in IDH mutant grade III and IV gliomas neither decreased the differentiation of DCs nor the functionality of differentiated DCs nor interfered with the processing or presentation of DC antigens. 2-HG did not inhibit T cell function indirectly through DC differentiation, rather it directly suppressed CD4^+^ and CD8^+^ T cells, activated Th1 and Th17, and regulated T cell (Treg) proliferation and cytokine generation in malignancies. IFN-γ production by activated T cells was inhibited in a dose-dependent manner, which inhibited the migration of T cells (40). In addition, 2-HG reduces antigen-presenting characteristics in macrophages, suppresses macrophage phenotype, and affects T-cell proliferation and effector cytokine production (46). 2-HG generated from tumors with IDH mutations can be taken up by T cells, the accumulation of intracellular 2-HG leads to increased apoptosis, decreased proliferation, and decreased Treg (47). Furthermore, high levels of 2-HG were found in T cells of IDH mutated AML patients, D-2HG caused HIF-1α protein instability, which led to a metabolic shift toward oxidative phosphorylation, an increase in regulatory T cell (Treg) frequency, and a decrease in Th17 polarization (48). It was also reported that NFAT transcription and polyamine synthesis were disturbed after 2-HG uptake, thus decreasing the ATP/ADP ratio and thereby inhibiting T cell activity and proliferation (49).

**Figure 2:**
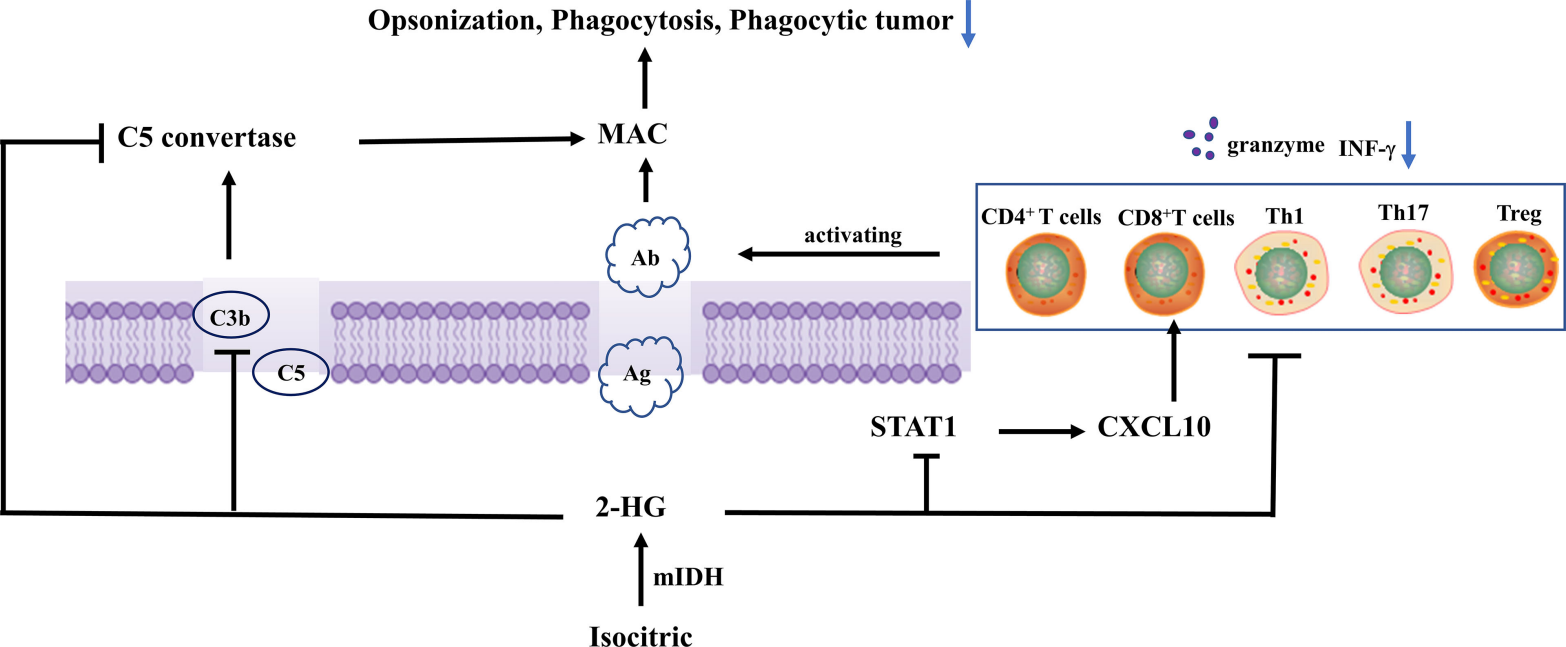
Isocitrate is metabolized to 2-hydroxyglutarate by mutant IDH, and 2-HG reduces C3 deposition on cells in a dose-dependent manner. In addition, it inhibits C3b (iC3b) opsonization and complement-mediated phagocytosis, and complement activation assembly of C5 convertases in the classical pathway, thereby inhibiting MAC-mediated tumor cell damage. 2-Hg inhibited the protein expression level of STAT1, thus decreasing chemokines related to type 1 (such as CXCL10), and affecting the aggregation of CD8+ T cells. in addition, it also directly inhibited the proliferation and cytokine production of CD4+ and CD8+ T cells, activated Th1, Th17, and Treg in tumors, and inhibited IFN-γ production by activating T cells in a dose-dependent manner, thereby inhibiting T cell migration.

Cytotoxic CD8^+^ T cells can recognize tumor-associated antigens in the presence of major histocompatibility complex (MHC) class I expressing tumors (50–52). Tumor-specific type 1 CD8^+^ T cells (mainly secreting IFN-γ) can effectively enter brain tumor sites through the type 1 chemokine CXCL10 and effectively kill tumor cells (53–56). Gary et al. (32) detected a lower level of type 1-related effector molecules, chemokines, and CD8^+^ T cells in IDH mutant gliomas after analyzing the clinical specimens and TCGA RNA-seq data. Their orthotopic syngeneic glioma model demonstrated that IDH1 R132H mutation suppressed the STAT1 protein expression *via* 2-HG, thus decreasing the type 1-associated chemokines such as CXCL10 and affecting CD8^+^ T cell aggregation (13) (**Figure 2**).

### Tumor-Derived Tiny Extracellular Vesicles and Immune Microenvironment

Tumor-derived tiny extracellular vesicles (TEX) contain a series of proteins, nucleic acids, and lipids that are similar in origin to tumors (57). TEX could transmit information from the tumor to cells near or far away from the tumor (including the bone marrow and lymphatic system), enabling cell-to-cell communication (58). To drive tumor growth, TEX can mediate juxtacrine, paracrine, and endocrine-like signals (59–62). TEX is an appealing candidate for regulating systemic tumor immune responses due to its physiologic origins and biodistribution features. The immunosuppressive effects of TEX are mediated by their immunosuppressive freight, and the intercellular distribution of these cargo components triggers pro-tumorigenic reprogramming of immune cells, which eventually promotes tumor growth (63–65).

Recent studies have shown that IDH mutant glioma cells produced more TEX than IDH wild-type glioma cells and were able to cross the blood-brain barrier to target anatomical sites (bone marrow, spleen) with abundant immune cells for phenotypic/functional regulation of circulating immune cells. Meanwhile, the expression of immune cell-related genes (ULBP1, ULBP3, RBP1, and CCL2) was decreased in IDH mutant glioma TEX, which were important regulators of immunosuppression and shape the immune cell composition of the systemic and local immune landscape (66).

## Immunotherapy of Isocitrate Dehydrogenase Mutant Glioma

Neurosurgical resection and adjuvant chemoradiation can prolong the survival of glioma patients, but recurrence occurs in most diffuse glioma cases, which largely limits the expected lifespan of the patients. Most currently available novel biological therapies have failed to significantly reduce the mortality and recurrence rate, which urges researchers to develop new therapeutic methods for glioma patients. Immunotherapies that target particular immunoregulatory molecules, known as ICIs and vaccine-mediated immunity, have demonstrated clinically meaningful effectiveness in a variety of tumor types, and have emerged as a distinct therapeutic strategy in cancer biology.

Immune checkpoints are a variety of immunosuppressive pathways in the immune system which are important for keeping self-tolerance and controlling the timing and amplitude of physiological immune responses in peripheral tissues to reduce collateral tissue injury (67). Tumor cells stimulate myeloid-derived suppressor cells to upregulate PD-L1 on the cell surface involvement in T cell immune checkpoints (68–71). Under normal circumstances, PD-L1 can bind to PD-1 on T cells and inhibit the activation of T cells, thereby avoiding the occurrence of autoimmune diseases. In tumors, after the combination of PD-L1 on the tumor cell membrane and PD-1, killer CD8+ T cells no longer recognize and kill tumor cells, which provides tumor cells with an opportunity to survive and develop. In gliomas, IDH mutant has been linked to reduced immunological checkpoint(PD-1, CTLA-4, LAG3, and IDO1) expression and immunosuppressive cell infiltration (72, 73). Compared with IDH-Os, IDH-As appear to be more responsive to checkpoint immunotherapy, one reason for the relatively poor anti-checkpoint immunotherapy of IDH-Os is its reduced expression of PD-L1 and other checkpoint molecules, another reason is higher levels of T cell rejection, promoting T cell dysfunction and immunotherapy resistance (15, 74, 75). Berghoff et al. observed that the IDH mutation status was the main factor affecting PD-L1 expression in diffuse gliomas, 2-hydroxyglutarate enhanced DNA methylation and suppresses PD-L1 expression (76–78). Because IDH mutant patients have lower PD-L1 expression, inhibitors of the PD1/PD-L1 immunological checkpoint are not recommended and other options should be explored (79). Some scholars have also proposed that targeted immunotherapy of IDH-mutant gliomas with decitabine can recover NKG2DLs expression and NK cell activation in experimental gliomas (32). In lung cancer and colorectal cancer, decitabine triggers tumor PD-L1 expression by inducing DNA hypomethylation, triggering an anticancer immune response, and remodeling the tumor microenvironment to improve the effect of PD-L1 immunotherapy (80, 81). We speculate that decitabine can also trigger PD-L1 expression in IDH-mutant glioma cells.

IDH is a promising target for immunization from an immunological standpoint since it is a tumor-specific latent neoantigen with significant homogeneity and penetration in all cancerous cells (82, 83). In glioma patients, R132H-mutated IDH1 is spontaneously processed, and an immunodominant epitope in the p123-142 region of MHC class II molecules is presented to CD4+ T cells, inducing spontaneous mutation-specific TH1 polarization and generation of mutation-specific antibodies. Schumacher et al. (43) discovered that IDH1R132H contained immunogenic epitopes suitable for mutation-specific vaccination, and inoculation of peptide vaccine IDH1 (R132H) P123-142 in homologous MHC humanized mice induced specific therapeutic T-helper cell responses and mutation-specific antibodies (84).

This notion is further supported by the study of Serena et al. (44), who constructed a mouse intracranial glioma model with IDH1R132H mutation and treated mIDH1-GL261 with mIDH1 peptide glioma mice. They found that the immune system could effectively target the R132H mutation and modify TME, increase the number of peripheral CD8+ T cells, IFN-γ production, and anti-IDH1 mutant antibodies, up-regulated intratumoral IFN-γ, granzyme-β, and perforin-1, and downregulated TGF-β2 and IL-10, thereby significantly prolonging survival of the mice. NOA-16 is the first IDH1R132H peptide vaccine in a multicenter stage I clinical study in humans. According to the latest report from this trial, more than 90% of patients developed vaccine-induced IDH1R132H-specific T cell reactions and peripheral T cell responses (85).

**Figure 3:**
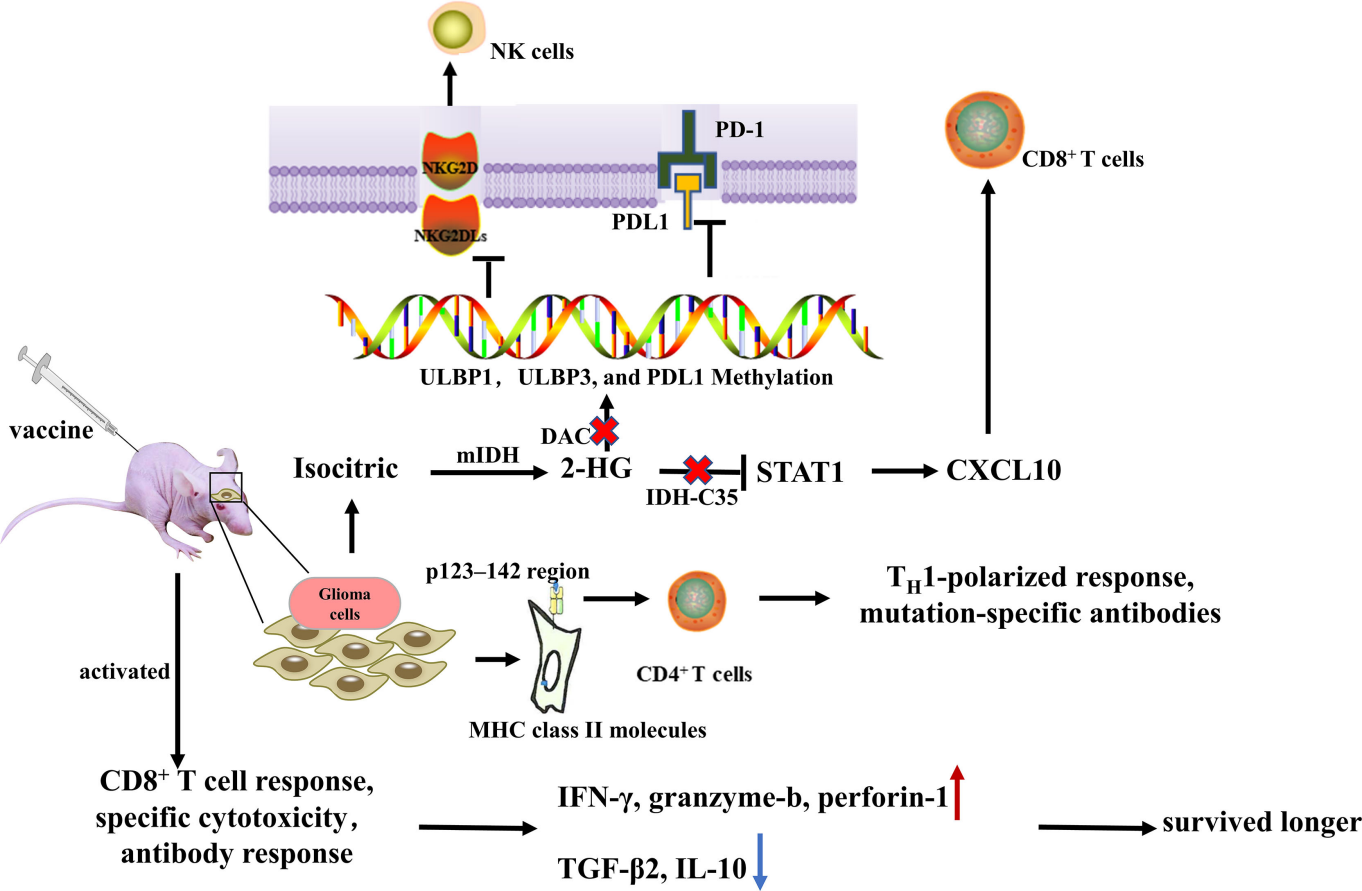
Administration of peptide vaccine IDH1 (R132H) induced specific therapeutic T-helper cell responses and mutation-specific antibodies. After mIDH1 peptide injection, the immune system can effectively target R132H mutation and modify the tumor microenvironment by increasing the number of peripheral CD8+ T cells, IFN-γ production and anti-IDH1 mutant antibodies, up-regulating intratumoral IFN- γ, granzyme-b, and perforin-1, and downregulating TGF-β2 and IL-10, thereby significantly prolonging survival of the mice. IDH-C35 reduces ULBP1, ULBP3, and PD-L1 DNA methylation levels and restores STAT1 to reverse CXCL10 and T cell accumulation.

R132H-IDH1 inhibitor (AGI-5198) blocked, in a dose-dependent manner, the ability of the mutant enzyme (mIDH1) to produce R-2-hydroxyglutarate (R-2HG), delayed growth and promoted differentiation of glioma cells (86). IDH-C35, a unique IDH1 inhibitor, can reverse the immunosuppressive effect of mutant IDH1. In immortalized NHAs and a homogeneous mouse glioma model, Gary et al. demonstrated that restoration of STAT1 with IDH-C35 reversed the reduction in CXCL10 and T cell accumulation, when combined with the vaccine, it could enhance anti-tumor immune function and the performance of peptide vaccines and improve asymptomatic survival of IDH-mutant glioma patients (13, 78). In addition, IDH-C35 can also reduce the level of PD-L1 DNA methylation, increase the expression of PD-L1 on mIDH1 glioma cells *in vivo*, reshape the tumor microenvironment, and improve the effect of PD-L1 immunotherapy (87). Therefore, IDH inhibitors should be used in conjunction with immunotherapy. Moreover, granulocyte colony-stimulating factor (G-CSF) released by mutIDH1 glioma stem-like cells promoted myeloid cell reprogramming, which improved the efficiency of immune-stimulatory gene therapy (88) (**Figure 3**).

## Conclusion

IDH1 is a reliable diagnostic and prognostic marker for identifying low-grade gliomas and distinguishing between secondary and primary GBM (89). IDH-mutant gliomas evade immune cell surveillance by reducing the immune-related cellular content and function of effector immune cells, downregulating NKG2D ligand expression, and overproducing 2-HG to inhibit complement pathways and T cell responses to interfere with immune surveillance and affect TME. The immune evasion ability of tumors provides an opportunity to harness immunotherapies against cancer cells.

Immunotherapies, particularly immune checkpoint inhibitors, have seen dramatic responses in various tumor types. Nevertheless, existing immunotherapies do not work on all malignancies, and even when they do, the results aren’t necessarily long-lasting. IDH-mutant gliomas have a unique immune microenvironment, 2-hydroxyglutarate is an oncogenic metabolite produced by mutant IDH with neurotoxic effects. Controlling 2-HG production and its effects may hold great promise for glioma therapy. Currently, the therapeutic efficacy of mutation-specific IDH inhibitors may partly depend on restoring the immune response in solid tumors by removing 2-HG. The combination of checkpoint blockade, decitabine, and IDH1R132H-specific vaccine may be more effective. Growing evidence certainly suggests that IDH mutations may play a broad role in glioma-associated immunosuppression. However, our understanding of the molecular mechanisms responsible for this unique immune microenvironment remains limited. For example, Why IDH-Os and IDH-As have different immune cell infiltration and PD-L1 expression? Does the localization of IDH1 in different places (cytoplasmic and peroxisome) affect immune cell infiltration and WHO grade? The research on IDH-mutant gliomas and the immunosuppressive mechanisms in the glioma microenvironment will help develop immunotherapy drugs and design new immunotherapies to target potential glioma therapeutic targets and kill tumor cells more efficiently.

## Author Contributions

DY, KY, and QL designed the paper and recommended a structure for the review. DY and WL wrote the initial draft and prepared figures. DY, KY, and QL completed the final preparation and editing of the manuscript. DY created the figures. All authors contributed to the article and approved the submitted version.

## Funding

This work was supported by Hainan Medical University Postgraduate Innovative Research Project Category B (HYYS2021B16)and the National Natural Science Foundation of China (GKJ200032). The funders had no role in the study design, data collection, analysis, decision to publish, or preparation of the manuscript.

## Conflict of Interest

The authors declare that the research was conducted in the absence of any commercial or financial relationships that could be construed as a potential conflict of interest.

## Publisher’s Note

All claims expressed in this article are solely those of the authors and do not necessarily represent those of their affiliated organizations, or those of the publisher, the editors and the reviewers. Any product that may be evaluated in this article, or claim that may be made by its manufacturer, is not guaranteed or endorsed by the publisher.

## References

[1] Louis DN Perry A Reifenberger G von Deimling A Figarella-Branger D Cavenee WK. The 2016 World Health Organization Classification of Tumors of the Central Nervous System: A Summary. Acta Neuropathol (2016) 131(6):803–20. doi: 10.1007/s00401-016-1545-1 27157931

[2] Reifenberger G Wirsching HG Knobbe-Thomsen CB Weller M. Advances in the Molecular Genetics of Gliomas - Implications for Classification and Therapy. Nat Rev Clin Oncol (2017) 14(7):434–52. doi: 10.1038/nrclinonc.2016.204 28031556

[3] Weller M Wick W Aldape K Brada M Berger M Pfister SM. Glioma. Nat Rev Dis Primers (2015) 1:15017. doi: 10.1038/nrdp.2015.17 27188790

[4] Venteicher AS Tirosh I Hebert C Yizhak K Neftel C Filbin MG. Decoupling Genetics, Lineages, and Microenvironment in Idh-Mutant Gliomas by Single-Cell Rna-Seq. Science (2017) 355(6332):eaai8478. doi: 10.1126/science.aai8478 28360267 PMC5519096

[5] Babikir H Wang L Shamardani K Catalan F Sudhir S MK A. Atrx Regulates Glial Identity and the Tumor Microenvironment in Idh-Mutant Glioma. Genome Biol (2021) 22(1):311. doi: 10.1186/s13059-021-02535-4 34763709 PMC8588616

[6] Yan H Parsons DW Jin G McLendon R Rasheed BA Yuan W. Idh1 and Idh2 Mutations in Gliomas. N Engl J Med (2009) 360(8):765–73. doi: 10.1056/NEJMoa0808710 PMC2820383 19228619

[7] Dang L White DW Gross S Bennett BD Bittinger MA Driggers EM. Cancer-Associated Idh1 Mutations Produce 2-Hydroxyglutarate. Nature (2009) 462(7274):739–44. doi: 10.1038/nature08617 PMC2818760 19935646

[8] Dang L Yen K Attar EC. Idh Mutations in Cancer and Progress Toward Development of Targeted Therapeutics. Ann Oncol (2016) 27(4):599–608. doi: 10.1093/annonc/mdw013 27005468

[9] D’Alessio A Proietti G Sica G Scicchitano BM. Pathological and Molecular Features of Glioblastoma and Its Peritumoral Tissue. Cancers (Basel) (2019) 11(4):469. doi: 10.3390/cancers11040469 PMC6521241 30987226

[10] Hanahan D Weinberg RA. Hallmarks of Cancer: The Next Generation. Cell (2011) 144(5):646–74. doi: 10.1016/j.cell.2011.02.013 21376230

[11] Bunse L Pusch S Bunse T Sahm F Sanghvi K Friedrich M. Suppression of Antitumor T Cell Immunity by the Oncometabolite (R)-2-Hydroxyglutarate. Nat Med (2018) 24(8):1192–203. doi: 10.1038/s41591-018-0095-6 29988124

[12] Amankulor NM Kim Y Arora S Kargl J Szulzewsky F Hanke M. Mutant Idh1 Regulates the Tumor-Associated Immune System in Gliomas. Genes Dev (2017) 31(8):774–86. doi: 10.1101/gad.294991.116 PMC5435890 28465358

[13] Kohanbash G DA C Shrivastav S BJ A Jahan N Mazor T. Isocitrate Dehydrogenase Mutations Suppress Stat1 and Cd8+ T Cell Accumulation in Gliomas. J Clin Invest (2017) 127(4):1425–37. doi: 10.1172/JCI90644 PMC5373859 28319047

[14] Makarevic A Rapp C Dettling S Reuss D Jungk C Abdollahi A. Increased Radiation-Associated T-Cell Infiltration in Recurrent Idh-Mutant Glioma. Int J Mol Sci (2020) 21(20):7801. doi: 10.3390/ijms21207801 PMC7590222 33096928

[15] Zhao B Xia Y Yang F Wang Y Wang Y Wang Y. Molecular Landscape of Idh-Mutant Astrocytoma and Oligodendroglioma Grade 2 Indicate Tumor Purity as an Underlying Genomic Factor. Mol Med (2022) 28(1):34. doi: 10.1186/s10020-022-00454-z 35287567 PMC8919570

[16] Zhang C Cheng W Ren X Wang Z Liu X Li G. Tumor Purity as an Underlying Key Factor in Glioma. Clin Cancer Res (2017) 23(20):6279–91. doi: 10.1158/1078-0432.CCR-16-2598 28754819

[17] Rollins BJ. Chemokines. Blood (1997) 90(3):909–28. doi: 10.1182/blood.V90.3.909 9242519

[18] Yoshie O Imai T Nomiyama H. Chemokines in Immunity. Adv Immunol (2001) 78:57–110. doi: 10.1016/S0065-2776(01)78002-9 11432208

[19] Guo RF Ward PA. Role of C5a in Inflammatory Responses. Annu Rev Immunol (2005) 23:821–52. doi: 10.1146/annurev.immunol.23.021704.115835 15771587

[20] Darling VR Hauke RJ Tarantolo S Agrawal DK. Immunological Effects and Therapeutic Role of C5a in Cancer. Expert Rev Clin Immunol (2015) 11(2):255–63. doi: 10.1586/1744666X.2015.983081 PMC4637164 25387724

[21] Sokratous G Polyzoidis S Ashkan K. Immune Infiltration of Tumor Microenvironment Following Immunotherapy for Glioblastoma Multiforme. Hum Vaccin Immunother (2017) 13(11):2575–82. doi: 10.1080/21645515.2017.1303582 PMC5703406 28362548

[22] Moretta A Bottino C Vitale M Pende D. Activating Receptors and Coreceptors Involved in Human Natural Killer Cell-Mediated Cytolysis. Annu Rev Immunol (2001) 19:197–223. doi: 10.1146/annurev.immunol.19.1.197 11244035

[23] Voskoboinik I Smyth MJ Trapani JA. Perforin-Mediated Target-Cell Death and Immune Homeostasis. Nat Rev Immunol (2006) 6(12):940–52. doi: 10.1038/nri1983 17124515

[24] Krzewski K Coligan JE. Human Nk Cell Lytic Granules and Regulation of Their Exocytosis. Front Immunol (2012) 3:335. doi: 10.3389/fimmu.2012.00335 23162553 PMC3494098

[25] Eagle RA Trowsdale J. Promiscuity and the Single Receptor: Nkg2d. Nat Rev Immunol (2007) 7(9):737–44. doi: 10.1038/nri2144 17673918

[26] V Groh TS. Immunobiology of Human Nkg2d and Its Ligands. Curr Top Microbiol Immunol (2006) 298:121–38. doi: 10.1007/3-540-27743-9_6 16329186

[27] Nausch N Cerwenka A. Nkg2d Ligands in Tumor Immunity. Oncogene (2008) 27(45):5944–58. doi: 10.1038/onc.2008.272 18836475

[28] Bauer S Groh V Wu J Steinle A Phillips JH Lanier LL. Activation of Nk Cells and T Cells by Nkg2d, a Receptor for Stress-Inducible Mica. Science (1999) 285(5428):727–9. doi: 10.1126/science.285.5428.727 10426993

[29] Pende D Rivera P Marcenaro S Chang C-C. Major Histocompatibility Complex Class I-Related Chain a and Ul16-Binding Protein Expression on Tumor Cell Lines of Different Histotypes: Analysis of Tumor Susceptibility to Nkg2d-Dependent Natural Killer Cell Cytotoxicity. Cancer Res (2002) 62(21):6178–86. 12414645

[30] Paulick MG Bertozzi CR. The Glycosylphosphatidylinositol Anchor: A Complex Membrane-Anchoring Structure for Proteins. Biochemistry (2008) 47(27):6991–7000. doi: 10.1021/bi8006324 18557633 PMC2663890

[31] Cosman D llberg JM Claire L. Sutherland, Wilson Chin, Richard Armitage. Ulbps, Novel Mhc Class I–Related Molecules, Bind to Cmv Glycoprotein Ul16 and Stimulate Nk Cytotoxicity Through the Nkg2d Receptor. Immunity (2001) 14(2):123–33. doi: 10.1016/s1074-7613(01)00095-4 11239445

[32] Zhang X Rao A Sette P Deibert C Pomerantz A Kim WJ. Idh Mutant Gliomas Escape Natural Killer Cell Immune Surveillance by Downregulation of Nkg2d Ligand Expression. Neuro Oncol (2016) 18(10):1402–12. doi: 10.1093/neuonc/now061 PMC5035522 27116977

[33] Ren F Zhao Q Huang L Zheng Y Li L He Q. The R132h Mutation in Idh1 Promotes the Recruitment of Nk Cells Through Cx3cl1/Cx3cr1 Chemotaxis and Is Correlated With a Better Prognosis in Gliomas. Immunol Cell Biol (2019) 97(5):457–69. doi: 10.1111/imcb.12225 30575118

[34] Luoto S Hermelo I Vuorinen EM Hannus P Kesseli J Nykter M. Computational Characterization of Suppressive Immune Microenvironments in Glioblastoma. Cancer Res (2018) 78(19):5574–85. doi: 10.1158/0008-5472.CAN-17-3714 29921698

[35] Castriconi R Daga A Dondero A Zona G Poliani PL Melotti A. Nk Cells Recognize and Kill Human Glioblastoma Cells With Stem Cell-Like Properties. J Immunol (2009) 182(6):3530–9. doi: 10.4049/jimmunol.0802845 19265131

[36] Praz F Lesavre P. Alternative Pathway of Complement Activation by Human Lymphoblastoid B and T Cell Lines. J Immunol (1983) 131(3):1396–9. 6604099

[37] Kurita M Matsumoto M Tsuji S Kawakami M Suzuki Y Hayashi H. Antibody-Independent Classical Complement Pathway Activation and Homologous C3 Deposition in Xeroderma Pigmentosum Cell Lines. Clin Exp Immunol (1999) 116(3):547–53. doi: 10.1046/j.1365-2249.1999.00923.x PMC1905298 10361249

[38] Rogers LM Veeramani S Weiner GJ. Complement in Monoclonal Antibody Therapy of Cancer. Immunol Res (2014) 59(1-3):203–10. doi: 10.1007/s12026-014-8542-z PMC4381956 24906530

[39] Ehlenberger AG Nussenzweig V. The Role of Membrane Receptors for C3b and C3d in Phagocytosis. J Exp Med (1977) 145(2):357–71. doi: 10.1084/jem.145.2.357 PMC2180606 833545

[40] Zhang L Sorensen MD Kristensen BW Reifenberger G McIntyre TM Lin F. D-2-Hydroxyglutarate Is an Intercellular Mediator in Idh-Mutant Gliomas Inhibiting Complement and T Cells. Clin Cancer Res (2018) 24(21):5381–91. doi: 10.1158/1078-0432.CCR-17-3855 PMC6214730 30006485

[41] Unruh D Schwarze SR Khoury L Thomas C Wu M Chen L. Mutant Idh1 and Thrombosis in Gliomas. Acta Neuropathol (2016) 132(6):917–30. doi: 10.1007/s00401-016-1620-7 PMC5640980 27664011

[42] Swann JB Smyth MJ. Immune Surveillance of Tumors. J Clin Invest (2007) 117(5):1137–46. doi: 10.1172/JCI31405 PMC1857231 17476343

[43] Ho PC Kaech SM. Reenergizing T Cell Anti-Tumor Immunity by Harnessing Immunometabolic Checkpoints and Machineries. Curr Opin Immunol (2017) 46:38–44. doi: 10.1016/j.coi.2017.04.003 28458087 PMC5554719

[44] Yang I Tihan T Han SJ Wrensch MR Wiencke J Sughrue ME. Cd8+ T-Cell Infiltrate in Newly Diagnosed Glioblastoma Is Associated With Long-Term Survival. J Clin Neurosci (2010) 17(11):1381–5. doi: 10.1016/j.jocn.2010.03.031 PMC3064460 20727764

[45] Tanaka A Sakaguchi S. Targeting Treg Cells in Cancer Immunotherapy. Eur J Immunol (2019) 49(8):1140–6. doi: 10.1002/eji.201847659 31257581

[46] van Dierendonck X de Goede KE Van den Bossche J. Idh-Mutant Brain Tumors Hit the Achilles’ Heel of Macrophages With R-2-Hydroxyglutarate. Trends Cancer (2021) 7(8):666–7. doi: 10.1016/j.trecan.2021.06.003 34183306

[47] Richardson LG Nieman LT Stemmer-Rachamimov AO Zheng XS Stafford K Nagashima H. Idh-Mutant Gliomas Harbor Fewer Regulatory T Cells in Humans and Mice. Oncoimmunology (2020) 9(1):1806662. doi: 10.1080/2162402X.2020.1806662 32923170 PMC7458656

[48] Bottcher M Renner K Berger R Mentz K Thomas S Cardenas-Conejo ZE. D-2-Hydroxyglutarate Interferes With Hif-1alpha Stability Skewing T-Cell Metabolism Towards Oxidative Phosphorylation and Impairing Th17 Polarization. Oncoimmunology (2018) 7(7):e1445454. doi: 10.1080/2162402X.2018.1445454 29900057 PMC5993507

[49] Friedrich M Bunse L Wick W Platten M. Perspectives of Immunotherapy in Isocitrate Dehydrogenase-Mutant Gliomas. Curr Opin Oncol (2018) 30(6):368–74. doi: 10.1097/CCO.0000000000000478 30102604

[50] Liau LM Black KL Martin NA Sykes SN Bronstein JM Jouben-Steele L. Treatment of a Glioblastoma Patient by Vaccination With Autologous Dendritic Cells Pulsed With Allogeneic Major Histocompatibility Complex Class I–Matched Tumor Peptides. Neurosurg Focus (2000) 9(6):e8. doi: 10.3171/foc.2000.9.6.9 16817691

[51] Prins RM Liau LM. Immunology and Immunotherapy in Neurosurgical Disease. Neurosurgery (2003) 53(1):144–52. doi: 10.1227/01.neu.0000068865.34216.3a 12823883

[52] Yang I Kremen TJ Giovannone AJ Paik E Odesa SK Prins RM. Modulation of Major Histocompatibility Complex Class I Molecules and Major Histocompatibility Complex–Bound Immunogenic Peptides Induced by Interferon- and Interferon- Treatment of Human Glioblastoma Multiforme. J Neurosurg (2004) 100(2):310–9. doi: 10.3171/jns.2004.100.2.0310 15086239

[53] Nishimura F Dusak JE Eguchi J Zhu X Gambotto A Storkus WJ. Adoptive Transfer of Type 1 Ctl Mediates Effective Anti-Central Nervous System Tumor Response: Critical Roles of Ifn-Inducible Protein-10. Cancer Res (2006) 66(8):4478–87. doi: 10.1158/0008-5472.CAN-05-3825 16618775

[54] Fujita M Zhu X Sasaki K Ueda R Low KL Pollack IF. Inhibition of Stat3 Promotes the Efficacy of Adoptive Transfer Therapy Using Type-1 Ctls by Modulation of the Immunological Microenvironment in a Murine Intracranial Glioma. J Immunol (2008) 180(4):2089–98. doi: 10.4049/jimmunol.180.4.2089 18250414

[55] Fujita M Zhu X Ueda R Sasaki K Kohanbash G Kastenhuber ER. Effective Immunotherapy Against Murine Gliomas Using Type 1 Polarizing Dendritic Cells–Significant Roles of Cxcl10. Cancer Res (2009) 69(4):1587–95. doi: 10.1158/0008-5472.CAN-08-2915 PMC5450639 19190335

[56] Fujita M Kohanbash G Fellows-Mayle W Hamilton RL Komohara Y Decker SA. Cox-2 Blockade Suppresses Gliomagenesis by Inhibiting Myeloid-Derived Suppressor Cells. Cancer Res (2011) 71(7):2664–74. doi: 10.1158/0008-5472.CAN-10-3055 PMC3075086 21324923

[57] Kucharzewska P Christianson HC Welch JE Svensson KJ Fredlund E Ringner M. Exosomes Reflect the Hypoxic Status of Glioma Cells and Mediate Hypoxia-Dependent Activation of Vascular Cells During Tumor Development. Proc Natl Acad Sci USA (2013) 110(18):7312–7. doi: 10.1073/pnas.1220998110 PMC3645587 23589885

[58] Banks WA Sharma P Bullock KM Hansen KM Ludwig N Whiteside TL. Transport of Extracellular Vesicles Across the Blood-Brain Barrier: Brain Pharmacokinetics and Effects of Inflammation. Int J Mol Sci (2020) 21(12):4407. doi: 10.3390/ijms21124407 PMC7352415 32575812

[59] Webber J Steadman R Mason MD Tabi Z Clayton A. Cancer Exosomes Trigger Fibroblast to Myofibroblast Differentiation. Cancer Res (2010) 70(23):9621–30. doi: 10.1158/0008-5472.CAN-10-1722 21098712

[60] Lopatina T Gai C Deregibus MC Kholia S Camussi G. Cross Talk Between Cancer and Mesenchymal Stem Cells Through Extracellular Vesicles Carrying Nucleic Acids. Front Oncol (2016) 6:125. doi: 10.3389/fonc.2016.00125 27242964 PMC4876347

[61] Ludwig N Yerneni SS Razzo BM Whiteside TL. Exosomes From Hnscc Promote Angiogenesis Through Reprogramming of Endothelial Cells. Mol Cancer Res (2018) 16(11):1798–808. doi: 10.1158/1541-7786.MCR-18-0358 30042174

[62] Razzo BM Ludwig N Hong CS Sharma P Fabian KP Fecek RJ. Tumor-Derived Exosomes Promote Carcinogenesis of Murine Oral Squamous Cell Carcinoma. Carcinogenesis (2020) 41(5):625–33. doi: 10.1093/carcin/bgz124 PMC7350555 31245809

[63] Chen G Huang AC Zhang W Zhang G Wu M Xu W. Exosomal Pd-L1 Contributes to Immunosuppression and Is Associated With Anti-Pd-1 Response. Nature (2018) 560(7718):382–6. doi: 10.1038/s41586-018-0392-8 PMC6095740 30089911

[64] Azambuja JH Ludwig N Yerneni S Rao A Braganhol E Whiteside TL. Molecular Profiles and Immunomodulatory Activities of Glioblastoma-Derived Exosomes. Neurooncol Adv (2020) 2(1):vdaa056. doi: 10.1093/noajnl/vdaa056 32642708 PMC7262743

[65] Azambuja JH Ludwig N Yerneni SS Braganhol E Whiteside TL. Arginase-1+ Exosomes From Reprogrammed Macrophages Promote Glioblastoma Progression. Int J Mol Sci (2020) 21(11):3990. doi: 10.3390/ijms21113990 PMC7312363 32498400

[66] Ludwig N Rao A Sandlesh P Yerneni SS Swain AD Bullock KM. Characterization of Systemic Immunosuppression by Idh Mutant Glioma Small Extracellular Vesicles. Neuro Oncol (2022) 24(2):197–209. doi: 10.1093/neuonc/noab153 34254643 PMC8804898

[67] Pardoll DM. The Blockade of Immune Checkpoints in Cancer Immunotherapy. Nat Rev Cancer (2012) 12(4):252–64. doi: 10.1038/nrc3239 PMC4856023 22437870

[68] Ribas A. Adaptive Immune Resistance: How Cancer Protects From Immune Attack. Cancer Discovery (2015) 5(9):915–9. doi: 10.1158/2159-8290.CD-15-0563 PMC4560619 26272491

[69] Alsaab HO Sau S Alzhrani R Tatiparti K Bhise K Kashaw SK. Pd-1 and Pd-L1 Checkpoint Signaling Inhibition for Cancer Immunotherapy: Mechanism, Combinations, and Clinical Outcome. Front Pharmacol (2017) 8:561. doi: 10.3389/fphar.2017.00561 28878676 PMC5572324

[70] Freeman GJ Long AJ Iwai Y Bourque K. Engagement of the Pd-1 Immunoinhibitory Receptor by a Novel B7 Family Member Leads to Negative Regulation of Lymphocyte Activation. J Exp Med (2000) 192(7):1027–34. doi: 10.1084/jem.192.7.1027 PMC2193311 11015443

[71] Keir ME Liang SC Guleria I Latchman YE Qipo A Albacker LA. Tissue Expression of Pd-L1 Mediates Peripheral T Cell Tolerance. J Exp Med (2006) 203(4):883–95. doi: 10.1084/jem.20051776 PMC2118286 16606670

[72] Goldman MJ Craft B Hastie M Repecka K McDade F Kamath A. Visualizing and Interpreting Cancer Genomics Data *Via* the Xena Platform. Nat Biotechnol (2020) 38(6):675–8. doi: 10.1038/s41587-020-0546-8 PMC7386072 32444850

[73] Gonzalez N Asad AS Gomez Escalante J Pena Agudelo JA Nicola Candia AJ Garcia Fallit M. Potential of Idh Mutations as Immunotherapeutic Targets in Gliomas: A Review and Meta-Analysis. Expert Opin Ther Targets (2021) 25(12):1045–60. doi: 10.1080/14728222.2021.2017422 34904924

[74] Joyce JA Fearon DT. T Cell Exclusion, Immune Privilege, and the Tumor Microenvironment. Science (2015) 348(6230):74–80. doi: 10.1126/science.aaa6204 25838376

[75] Voabil P de Bruijn M Roelofsen LM Hendriks SH Brokamp S van den Braber M. An Ex Vivo Tumor Fragment Platform to Dissect Response to Pd-1 Blockade in Cancer. Nat Med (2021) 27(7):1250–61. doi: 10.1038/s41591-021-01398-3 34239134

[76] Berghoff AS Kiesel B Widhalm G Wilhelm D Rajky O Kurscheid S. Correlation of Immune Phenotype With Idh Mutation in Diffuse Glioma. Neuro Oncol (2017) 19(11):1460–8. doi: 10.1093/neuonc/nox054 PMC5737620 28531337

[77] Mu L Long Y Yang C Jin L Tao H Ge H. The Idh1 Mutation-Induced Oncometabolite, 2-Hydroxyglutarate, May Affect DNA Methylation and Expression of Pd-L1 in Gliomas. Front Mol Neurosci (2018) 11:82. doi: 10.3389/fnmol.2018.00082 29643764 PMC5882817

[78] Pirozzi CJ Yan H. The Implications of Idh Mutations for Cancer Development and Therapy. Nat Rev Clin Oncol (2021) 18(10):645–61. doi: 10.1038/s41571-021-00521-0 34131315

[79] Kayabolen A Yilmaz E Bagci-Onder T. Idh Mutations in Glioma: Double-Edged Sword in Clinical Applications? Biomedicines (2021) 9(7):799. doi: 10.3390/biomedicines9070799 34356864 PMC8301439

[80] Huang KC Chiang SF Chen WT Chen TW Hu CH Yang PC. Decitabine Augments Chemotherapy-Induced Pd-L1 Upregulation for Pd-L1 Blockade in Colorectal Cancer. Cancers (Basel) (2020) 12(2):462. doi: 10.3390/cancers12020462 PMC7072566 32079180

[81] Lai Q Wang H Li A Xu Y Tang L Chen Q. Decitibine Improve the Efficiency of Anti-Pd-1 Therapy *Via* Activating the Response to Ifn/Pd-L1 Signal of Lung Cancer Cells. Oncogene (2018) 37(17):2302–12. doi: 10.1038/s41388-018-0125-3 29422611

[82] Capper D Weissert S Balss J Habel A Meyer J Jager D. Characterization of R132h Mutation-Specific Idh1 Antibody Binding in Brain Tumors. Brain Pathol (2010) 20(1):245–54. doi: 10.1111/j.1750-3639.2009.00352.x PMC8094636 19903171

[83] Watanabe T Nobusawa S Kleihues P Ohgaki H. Idh1 Mutations Are Early Events in the Development of Astrocytomas and Oligodendrogliomas. Am J Pathol (2009) 174(4):1149–53. doi: 10.2353/ajpath.2009.080958 PMC2671348 19246647

[84] Schumacher T Bunse L Pusch S Sahm F Wiestler B Quandt J. A Vaccine Targeting Mutant Idh1 Induces Antitumour Immunity. Nature (2014) 512(7514):324–7. doi: 10.1038/nature13387 25043048

[85] Platten M Bunse L Wick A Bunse T Le Cornet L Harting I. A Vaccine Targeting Mutant Idh1 in Newly Diagnosed Glioma. Nature (2021) 592(7854):463–8. doi: 10.1038/s41586-021-03363-z PMC8046668 33762734

[86] Rohle D Popovici-Muller J Palaskas N Turcan S Grommes C Campos C. An Inhibitor of Mutant Idh1 Delays Growth and Promotes Differentiation of Glioma Cells. Science (2013) 340(6132):626–30. doi: 10.1126/science.1236062 PMC3985613 23558169

[87] Kadiyala P Carney SV Gauss JC Garcia-Fabiani MB Haase S Alghamri MS. Inhibition of 2-Hydroxyglutarate Elicits Metabolic Reprogramming and Mutant Idh1 Glioma Immunity in Mice. J Clin Invest (2021) 131(4):e139542. doi: 10.1172/JCI139542 PMC7880418 33332283

[88] Alghamri MS McClellan BL Avvari RP Thalla R Carney S Hartlage MS. G-Csf Secreted by Mutant Idh1 Glioma Stem Cells Abolishes Myeloid Cell Immunosuppression and Enhances the Efficacy of Immunotherapy. Sci Adv (2021) 7(40):eabh3243. doi: 10.1126/sciadv.abh3243 34586841 PMC8480930

[89] Xu W Yang H Liu Y Yang Y Wang P Kim SH. Oncometabolite 2-Hydroxyglutarate Is a Competitive Inhibitor of Alpha-Ketoglutarate-Dependent Dioxygenases. Cancer Cell (2011) 19(1):17–30. doi: 10.1016/j.ccr.2010.12.014 21251613 PMC3229304

